# Intervention With Autologous Platelet‐Containing Plasma for Immature Atrophic Facial Scars: A Quantitative Case Study

**DOI:** 10.1002/ccr3.73236

**Published:** 2026-08-07

**Authors:** Virgilio Blandón, Sofia González, Hildemar Baltodano

**Affiliations:** ^1^ Faculty of Medicine Universidad Americana (UAM) Managua Nicaragua

**Keywords:** cicatrix, facial injuries, Fiji, ImageJ, injections, platelet‐rich plasma

## Abstract

Intradermal autologous platelet‐containing plasma (ACP) was applied to immature atrophic scars during early remodeling. Results showed improvements in depth, surface area, and texture over time. Although effects may overlap with natural healing, ACP appears safe, minimally invasive, and deserving of further controlled studies to clarify its role in dermal remodeling.

## Graphical abstract

Current clinical practice for atrophic scars typically delays intervention until scar stabilization, usually 6–12 months after injury. This case presents a young male with an immature atrophic facial scar who received intradermal autologous platelet‐containing plasma (ACP) 2 months after injury. Four treatment sessions were administered at 2‐week intervals. Over 6 months of follow‐up, objective digital planimetry using ImageJ with internal anatomical calibration documented progressive reductions in scar surface area and depth. Concurrently, POSAS 3.0 observer and patient scores showed consistent improvements in erythema, texture, and overall scar quality. The procedure was well tolerated, with no adverse events reported during treatment or follow‐up. While the contribution of natural scar maturation cannot be entirely excluded, the observed improvements in a depressed atrophic scar, a phenotype where spontaneous elevation is considered uncommon, suggest that early intervention during the immature phase may be associated with measurable benefits. These findings support the need for controlled studies to further explore the therapeutic potential of ACP in early atrophic scar management.

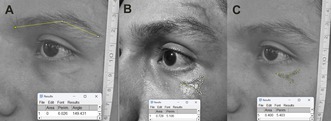

## 1 Introduction

Post‐traumatic facial scarring represents a significant clinical challenge in dermatologic surgery, with the atrophic variant posing particular therapeutic difficulties [1, 2, 3]. The initial months following re‐epithelialization constitute a critical remodeling phase characterized by dynamic and often unpredictable biological processes, during which the natural resolution of inflammation and the reorganization of collagen architecture can lead to substantial spontaneous improvement [4]. This inherent biological variability, the complex influence of which has been highlighted through hierarchical Bayesian analysis of wound biomechanics using a murine data‐calibrated in silico model [5], complicates the assessment of any therapeutic intervention applied during this formative period.

Current therapeutic paradigms for atrophic scarring predominantly target mature, established scars, typically after a stabilization period of 6–12 months [1, 2]. Conventional modalities such as laser resurfacing, dermal fillers, and microneedling can be effective but are often costly, invasive, and primarily address established scar features rather than actively guiding the initial healing process. Growing evidence supports the potential for intervention during the immature phase (the period prior to stabilization), with systematic reviews demonstrating that laser‐based treatments initiated within the first 3 months post‐wounding can significantly improve scar outcomes [6]. Among autologous options, fat transplantation has shown promise but carries substantially higher complication rates due to its invasive nature [7], highlighting the clinical need for effective yet safer immature‐stage treatment options.

Analogous to its well‐documented, synergistic role in enhancing the survival and integration of autologous fat grafts [8], autologous platelet‐containing plasma (ACP) offers a safe, minimally invasive approach for soft tissue repair. Building on this principle of biostimulation, ACP intervention during the immature phase of a cutaneous wound has been proposed as a strategy to act synergistically with endogenous healing processes. Concentrated platelets release a range of growth factors from their α‐granules, such as platelet‐derived growth factor (PDGF), transforming growth factor beta (TGF‐β), vascular endothelial growth factor (VEGF), and epidermal growth factor (EGF), while the plasma fraction provides additional mediators like insulin‐like growth factor 1 (IGF‐1), which are not highly stored in platelets [9]. Together, these bioactive elements regulate key cellular processes throughout the different stages of tissue repair: they recruit and activate inflammatory cells, stimulate fibroblast proliferation and extracellular matrix production during the proliferative phase, and promote angiogenesis as well as matrix remodeling in the maturation phase [9]. After activation, the protein‐rich fibrin matrix that forms functions both as a temporary scaffold and as a system for the sustained release of embedded cells and growth factors, extending the biological effect beyond the initial application [9]. This combined mechanism, which integrates the delivery of multiple growth factors with a controlled release over time, supports the rationale for using ACP during the early and biologically active phase of scar formation.

Despite growing interest, our recent scoping synthesis of the available clinical literature highlights a marked scarcity of studies specifically addressing ACP and platelet‐rich plasma (PRP) usage in immature post‐traumatic scars, with most reports limited to small case series, heterogeneous protocols, and insufficient product characterization [10]. This limited evidence base also lacks robust longitudinal data employing objective and quantitative outcome measures, which are essential to reduce observational bias and enable meaningful comparisons, particularly given the difficulty of distinguishing true therapeutic effects from spontaneous maturation during the immature phase.

We present the case of a young male with post‐traumatic immature facial scarring, defined as scars in the early remodeling phase that remain biologically active with ongoing collagen and extracellular matrix reorganization [11]. This report provides a longitudinal assessment using both patient‐reported outcomes and objective digital image analysis. Although the single‐case design cannot disentangle the specific contribution of ACP from natural scar maturation, the intervention was well tolerated, with no adverse events, and was associated with observable clinical improvement over time. The primary objective is not to establish definitive efficacy, but to quantitatively document scar evolution under ACP exposure during the immature phase, supporting procedural safety and providing hypothesis‐generating data in an area of limited and heterogeneous clinical evidence.

## 2 Case History/Examination

**FIGURE 1:**
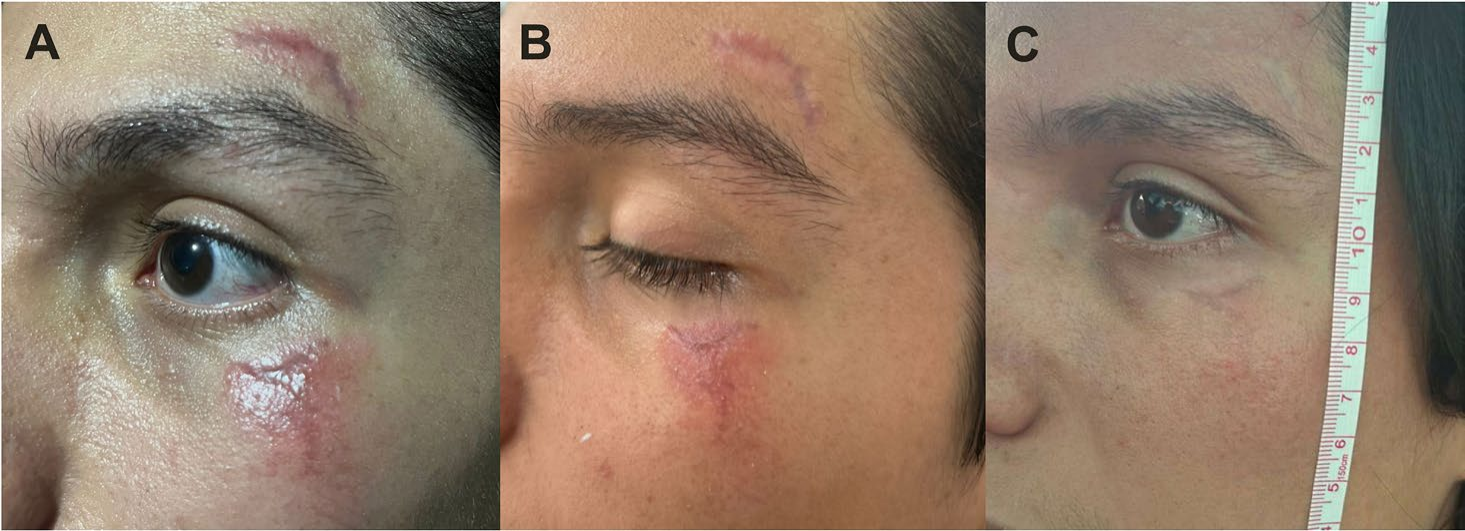
Composite image of the left malar and frontal regions demonstrating the scars at three distinct phases. To facilitate comparison and ensure patient de‐identification, original photographs were cropped and aligned using Photopea software without altering primary image data such as lighting or contrast. (A) Baseline evaluation showing immature atrophic scars with marked erythema and contour depression 2 months post‐injury. (B) Clinical appearance following the completion of four autologous platelet‐containing plasma (ACP) treatment sessions. (C) Six‐month follow‐up demonstrating improvement in skin texture, restoration of surface contour, and reduction in scar‐associated erythema.

A 20‐year‐old man presented with facial scarring on the left hemifacial region 2 months after suture removal, following injuries sustained in a motorcycle accident. The initial laceration, involving the left malar area, had undergone surgical revision with primary closure. At baseline evaluation, two distinct atrophic scars were observed: one malar (1.82 × 1.22 cm) and one frontal (2.70 × 0.75 cm), both exhibiting marked erythema, contour depression, and textural irregularity consistent with the immature phase (Figure 1).

## 3 Differential Diagnosis, Investigations, and Treatment

The clinical presentation was consistent with immature post‐traumatic atrophic facial scars in the early remodeling phase, based on the history of recent trauma, primary closure, and characteristic clinical features including erythema, surface depression, and textural irregularity. Given the clear traumatic etiology and typical morphological appearance, alternative diagnoses such as acne‐related scarring, inflammatory dermatoses, or primary lipoatrophic disorders were not considered likely, and no additional diagnostic investigations were required. Clinical evaluation and photographic documentation were considered sufficient to guide management.

Given the visible atrophy and patient concern regarding cosmetic appearance, a decision was made to proceed with autologous ACP therapy to enhance dermal remodeling. For each treatment session, 15 mL of venous blood was drawn into two 5 mL sodium citrate tubes. The blood was processed via single‐spin centrifugation at 2500 RPM (700 g with a 100 mm radius rotor) for 12 min at 24°C. To maximize platelet concentration and compensate for the inability to perform a cell count, a standardized volume of 1.0 mL of the deepest plasma fraction, located immediately above the buffy coat, was selectively aspirated from each tube. This approach was designed to ensure a high platelet yield by harvesting the most concentrated portion of the plasma column [12]. This yielded 2.0 mL of ACP per session; notably, platelet concentration was not quantified, representing a methodological limitation that precludes precise standardization or direct comparison with other studies. Prior to injection, platelet activation was induced by adding 10% calcium gluconate in a 1:10 ratio (0.1 mL calcium per 1.0 mL of plasma). This exogenous activation effectively neutralizes the anticoagulant effect of sodium citrate and ensures a standardized, immediate peak of growth factor availability [13], which is critical for maximizing the predictability of the regenerative response and overcoming the potential variability in endogenous activation within fibrotic or compromised tissue [14]. A fixed 1.0 mL aliquot of the activated plasma was allocated for injection into each of the two documented study scars.

A series of four treatments was administered at 2‐week intervals. Under topical anesthesia with 2.5% lidocaine gel, 1.0 mL of ACP was injected per scar using a 30‐gage needle, amounting to a total of 2.0 mL per session and a cumulative dose of 8.0 mL across the four‐4 protocol (4.0 mL per scar). Injections were performed at a 15‐degree angle to target the mid‐to‐deep reticular dermis. Injection depth was standardized by monitoring for transient blanching and visible elevation along the entire length of the depressed scar. The product was deposited using a linear threading technique until consistent tissue turgor was achieved throughout. The procedure was well tolerated, causing only mild and transient discomfort during needle insertion. No adjunctive therapies were administered during the observation period; the patient was only advised to use sunscreen regularly.

## 4 Conclusion and Results (Outcome and Follow‐Up)

Longitudinal assessment employed both observer‐ and patient‐reported metrics. The Observer and Patient components of the Patient and Observer Scar Assessment Scale (POSAS) 3.0 demonstrated consistent improvement for both scars [15]. The malar scar scores improved by 39.1% for the Observer and 60% for the Patient, while the frontal scar scores improved by 33.3% for the Observer and 55.2% for the Patient, reflecting parallel trends in perceived scar quality over 6 months (Table 1).

**TABLE 1:** Longitudinal scar assessment metrics.

Parameter	Baseline	Post‐treatment	6‐month follow‐up	Relative change (%)
Malar scar POSAS 3.0 Observer Score (7–35)	23	18	14	39.1
Malar scar POSAS 3.0 Patient Score (16–80)	45	24	18	60
Frontal scar POSAS 3.0 Observer Score (9–45)	24	19	16	33.3
Frontal scar POSAS 3.0 Patient Score (17–85)	38	20	17	55.2
Malar scar area (cm^2^)	0.728	0.546	0.400	45.0
Malar scar depth (cm)	0.40	0.15	0.10	75.0
Frontal scar area (cm^2^)	1.300	0.795	0.650	50.0
Frontal scar depth (cm)	0.100	0.060	0.040^a^	60.0

*Note:* Clinical and quantitative outcomes of malar and frontal scars over 6 months, showing consistent improvement in POSAS 3.0 scores and scar dimensions. Relative change (%) reflects the percentage reduction from baseline to 6‐month follow‐up, calculated as: [(Baseline − Follow‐up)/Baseline] × 100. All treatments were well‐tolerated.

^a^ Frontal scar depth at 6 months was measured approximately; the value (0.040 cm) is near the minimal detectable limit, but improvement was confirmed visually and quantitatively.

**FIGURE 2:**
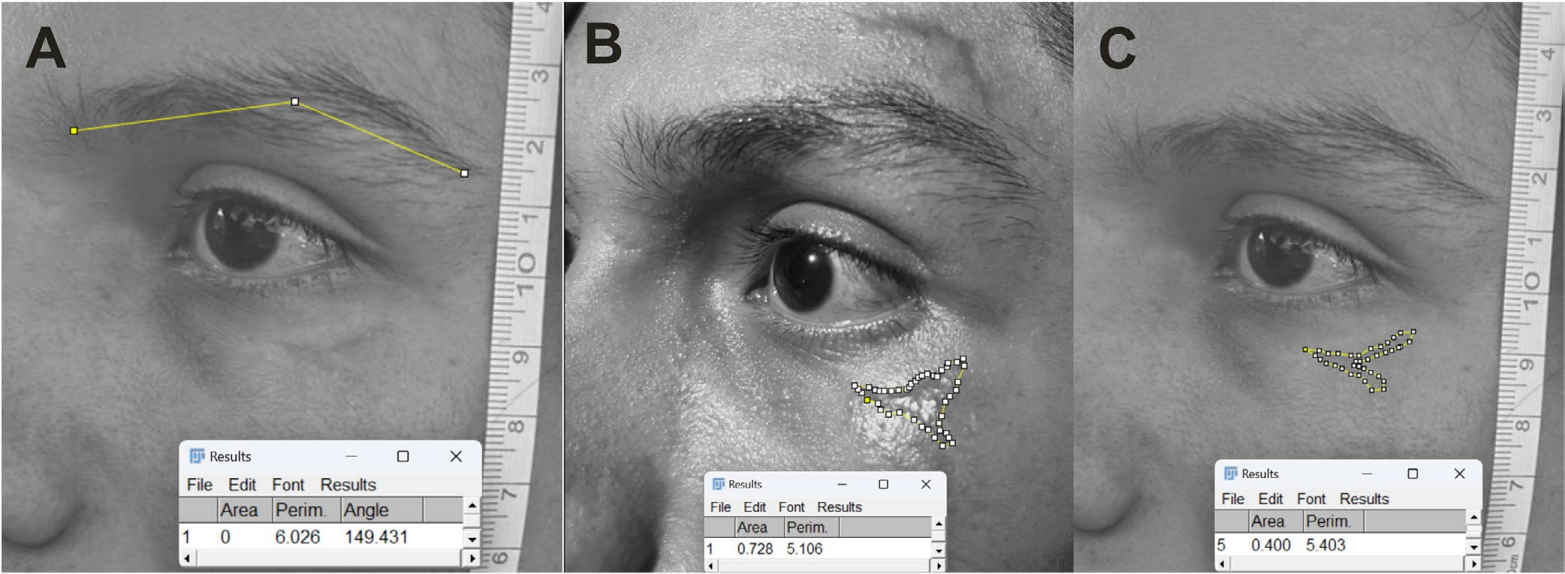
Clinical photographs were converted to grayscale to minimize visual distractions from minor lighting variations between sessions. (A) Spatial calibration using the patient's left eyebrow (6.0 cm) as an internal anatomical reference to establish the pixel‐to‐centimeter ratio. (B) Baseline evaluation and measurement of the malar scar area (0.728 cm^2^). (C) Six‐month follow‐up measurement showing a reduction in scar surface area (0.400 cm^2^). To facilitate direct visual comparison, the images were aligned and cropped in Photopea to focus on the regions of interest without altering the original morphological features.

Quantitative assessment included manual measurements of scar length, width, and depth, along with digital analysis using ImageJ (Fiji) with photographs captured in a series at the same clinical site. To improve consistency across the longitudinal follow‐up, efforts were made to maintain the patient in the same position and orientation for each session, ensuring a standardized resting facial expression to minimize eyebrow displacement due to muscle contraction. Prior to digital analysis, all photographs were converted to grayscale to mitigate differences in illumination between sessions. Digital planimetry was performed by a single assessor, who utilized the “Polygon Selection” tool to manually delineate the scar boundaries in triplicate for each time point to ensure intra‐rater reliability. ImageJ was used exclusively for two‐dimensional surface area measurements (cm^2^) and was not employed for the assessment of scar depth; therefore, it does not capture the true three‐dimensional volume or surface topography. Spatial calibration was based on the total length of the patient's left eyebrow (6.0 cm) as a stable internal anatomical reference. This measurement was manually verified with a physical ruler at each clinical session. Using the “Straight Line” tool, this 6.0 cm reference was traced to define the pixel‐to‐centimeter ratio via the “Set Scale” function, thereby compensating for variations in camera distance, zoom, or patient positioning between photographic sessions inherent to the clinical setting. An illustrative example of this measurement process and the resulting area comparisons is shown in ImageJ screenshots (Figure 2).

Quantitative analysis suggested gradual improvements across all parameters following the four ACP treatments. The malar scar area decreased from 0.728 to 0.546 cm^2^, with a depth reduction from 0.40 to 0.15 cm. The frontal scar area decreased from 1.30 to 0.795 cm^2^, while its depth was reduced from a baseline of 0.10 cm to a residual 0.04 cm. This measurement approached the minimal detectable limit and confirmed that the residual depression reached the level of the surrounding skin. Serial photographs appeared to support these observations, showing some restoration of surface contour and improvement in texture. Changes in erythema and vascularity were also reflected in the POSAS 3.0 scores, with a general trend toward lower observer‐ and patient‐reported scores over 6 months (see Tables S1–S4). No adverse events were reported during treatment or follow‐up.

## 5 Discussion

This case provides preliminary proof‐of‐concept observations regarding the clinical evolution of immature post‐traumatic facial scars monitored during intradermal ACP administration. Improvements were observed in both patient‐ and observer‐reported POSAS scores, as well as in scar surface area measured digitally, over 6 months. Notably, these improvements occurred in depressed atrophic scars, where spontaneous elevation to near‐normal skin level is considered uncommon even during the active remodeling phase [16]. Normal wound remodeling involves gradual replacement of type III collagen with type I collagen and reorganization of the extracellular matrix, but full restoration of dermal volume or surface contour is infrequently documented [16, 17].

To our knowledge, this appears to be among the first reports describing the use of ACP as monotherapy for an immature post‐traumatic facial scar [10]. The clinical and patient‐reported observations were complemented by longitudinal digital analysis with ImageJ (Fiji) to provide objective measurements of changes in scar surface area. The observed progressive improvements in both malar and frontal scars may be consistent with a potential influence of ACP on scar appearance and quality; however, the underlying biological mechanisms remain uncertain, and a contribution from natural scar maturation cannot be excluded. Treating immature scars presents specific challenges, primarily the difficulty of distinguishing therapeutic effects from spontaneous biological maturation during the first 6 months of healing [4, 5]. A significant knowledge gap remains regarding the optimal timing for ACP intervention during this active remodeling phase, as most existing literature lacks robust longitudinal data employing objective and quantitative outcome measures [10]. Consequently, identifying the precise contribution of ACP versus natural tissue resolution remains a primary clinical uncertainty. As noted earlier, while the inflammatory component (erythema) and texture often resolve spontaneously, the degree of volumetric filling observed remains an atypical feature of natural remodeling [16, 17], highlighting the importance of standardized imaging to establish definitive efficacy during this formative period.

Previous evidence from platelet‐rich plasma interventions suggests that ACP could deliver growth factors to the reticular dermis, potentially influencing fibroblast activity, collagen deposition, and extracellular matrix organization [18]. This is conceptually supported by interventions promoting extracellular matrix organization, including platelet‐rich plasma‐based scaffolds and biomimetic materials, which can guide cellular orientation and tissue regeneration in skin [19, 20]. Histopathological studies on mature atrophic post‐traumatic scars demonstrate that PRP can stimulate collagen deposition and dermal remodeling, even in stable scars unlikely to improve spontaneously [21]. While the biological context of mature scars differs due to established fibrosis, these studies provide a necessary baseline to understand PRP's regenerative potential. Similarly, preliminary clinical evidence from case reports and small series suggests that intervention during the immature phase with PRP or ACP may improve scar appearance and quality in post‐traumatic and burn‐related scars, primarily as an adjunctive therapy, by enhancing vascularity, collagen deposition, and matrix organization [22, 23, 24]. Collectively, these findings support the hypothesis that ACP could contribute to favorable changes in scar structure, although direct causal effects in immature scars remain unproven.

The simplicity and minimally invasive nature of the single‐spin centrifugation and intradermal injection protocol further support its clinical applicability. Although platelet concentration was not quantified, careful harvest of the deepest plasma fraction likely provided sufficient biological activity to influence remodeling [12]. No adverse events were observed, reinforcing the safety of this intervention.

It is important to recognize that this report represents a preliminary proof‐of‐concept based on a single case, and natural scar maturation cannot be entirely excluded as a contributing factor. Additional limitations include the lack of platelet quantification, absence of a control scar, and limited generalizability to other patients or scar types. Furthermore, while digital planimetric analysis allowed objective longitudinal comparison, the image acquisition was performed under clinical conditions rather than a strictly standardized photographic environment. Although efforts were made to maintain consistent patient positioning and spatial calibration was performed using internal anatomical references, minor variations in lighting and camera angle between sessions may exist. Crucially, 2D planimetry measurements from photographs do not capture true three‐dimensional surface area or volume. In the context of atrophic scars, where clinical improvement often involves the volumetric filling of dermal defects (reduction in depth) rather than just a contraction of the perimeter, our 2D approach may underestimate the total structural remodeling achieved.

Although the observed improvements in scar depth and contour are notable, they cannot be definitively distinguished from the effects of spontaneous remodeling during the immature phase. Taken together, these observations provide a preliminary clinical signal consistent with the hypothesis that ACP intervention may influence scar architecture during early healing. However, the findings should be interpreted with caution, and further studies involving larger cohorts, 3D volumetric imaging protocols, and controlled comparisons are necessary to clarify the specific contribution of ACP beyond natural scar maturation.

In conclusion, this case suggests that intradermal ACP injections during the immature phase of post‐traumatic facial scars may be associated with measurable improvements in scar depth, surface contour, and texture. While natural scar maturation cannot be entirely excluded, the observed clinical and quantitative outcomes suggest that ACP merits further investigation as a potentially safe and minimally invasive adjunctive approach for early scar remodeling. However, larger controlled studies are necessary to confirm these preliminary findings and optimize treatment protocols.

## Author Contributions

**Virgilio Blandón:** conceptualization, data curation, formal analysis, funding acquisition, investigation, methodology, project administration, resources, software, supervision, validation, visualization, writing – original draft, writing – review and editing. **Sofia González:** conceptualization, data curation, formal analysis, investigation, methodology, project administration, resources, software, validation, visualization, writing – original draft, writing – review and editing. **Hildemar Baltodano:** data curation, formal analysis, investigation, methodology, project administration, resources, software, validation, visualization, writing – review and editing.

## Funding

The authors have nothing to report.

## Ethics Statement

The authors have nothing to report.

## Consent

Written informed consent was obtained from the patient for both the clinical procedure and publication of this case report, including any accompanying images. The original consent form is on file and will be provided to the Publisher upon request.

## Conflicts of Interest

The authors declare no conflicts of interest.

## Supporting information


**Table S1:** POSAS 3.0 Observer Generic Scale: Itemized Assessment for the Malar Scar.
**Table S2:** POSAS 3.0 patient generic scar: Itemized Assessment for the Malar Scar.
**Table S3:** POSAS 3.0 observer linear scar: Itemized Assessment for the frontal scar.
**Table S4:** POSAS 3.0 patient linear scar: Itemized Assessment for the frontal scar.

## Data Availability

All data supporting this case report are presented within the article and its figures. Raw photographs are not publicly available to protect patient privacy. Anonymized measurement data are available from the corresponding author upon reasonable request.
