# Supplementary material for: Intervention With Autologous Platelet‐Containing Plasma for Immature Atrophic Facial Scars: A Quantitative Case Study
Source: Clin Case Rep. 2026 Aug 7;14(8):e73236. doi: 10.1002/ccr3.73236 (PMC13451649; doi:10.1002/ccr3.73236)
Supplement: Supplementary file 1 [file CCR3-14-e73236-s001.docx]

| **Items (Scale 1-5)** | **Baseline** | **Post-Treatment** | **Follow-up (6 months)** |
| --- | --- | --- | --- |
| Pigmentation | 4 (Severely/ Mixed) | 3 (Moderately/ Mixed) | 2 (Minimally/ Hyperpigmentation) |
| Vascularity | 4 (Severely/ Red) | 3 (Moderately/ Red) | 2 (Minimally/ Pink) |
| Surface level | 4 (Severely/ Depressed) | 3 (Moderately/ Depressed) | 2 (Minimally/ Depressed) |
| Surface texture | 4 (Severely/ Irregular) | 3 (Moderately/ Irregular) | 2 (Minimally/ Irregular) |
| Firmness | 3 (Moderately) | 2 (Minimally) | 2 (Minimally) |
| Adherence | 2 (Minimally) | 2 (Minimally) | 2 (Minimally) |
| Tension | 2 (Minimally) | 2 (Minimally) | 2 (Minimally) |
|  |  |  |  |
| **TOTAL (Sum 1-7)** | **23 / 35** | **18 / 35** | **14 / 35** |

**S1 Table. POSAS 3.0 Observer Generic Scale: Itemized Assessment for the Malar Scar.**

**S1 Table.** Assessment of malar scar characteristics using the POSAS 3.0 observer scale at baseline, post-treatment, and 6-month follow-up. Scores for each item range from 1 (normal skin) to 5 (severely affected), with total scores calculated as the sum of the seven items (maximum 35).

**S2 Table. POSAS 3.0 patient generic scar: Itemized Assessment for the Malar Scar.**

| **Items (Scale 1-5)** | **Baseline** | **Post-Treatment** | **Follow-up** |
| --- | --- | --- | --- |
| Colour (difference) | 4 (Very) | 2 (Little) | 2 (Little) |
| Shininess | 3 (Moderately) | 2 (Little) | 1 (Not) |
| Raised or Sunken (difference) | 4 (Very) | 1 (Not) | 1 (Not) |
| Hardness | 2 (Little) | 2 (Little) | 1 (Not) |
| Surface Irregularity | 5 (Extremely) | 3 (Moderately) | 2 (Little) |
| Sensitive to touch | 2 (Little) | 1 (Not) | 1 (Not) |
| Less sensitive (numbness) | 3 (Moderately) | 1 (Not) | 1 (Not) |
| Painful | 1 (Not) | 1 (Not) | 1 (Not) |
| Shooting sensation | 2 (Little) | 2 (Little) | 1 (Not) |
| Burning sensation | 1 (Not) | 1 (Not) | 1 (Not) |
| Itching | 2 (Little) | 1 (Not) | 1 (Not) |
| Tingling / Pins and needles | 3 (Moderately) | 1 (Not) | 1 (Not) |
| Tightness at rest | 3 (Moderately) | 2 (Little) | 1 (Not) |
| Tightness with movement | 4 (Very) | 2 (Little) | 1 (Not) |
| Fragility (easy breakdown) | 3 (Moderately) | 1 (Not) | 1 (Not) |
| Dryness | 3 (Moderately) | 1 (Not) | 1 (Not) |
| **TOTAL (Sum 1-16)** | **45 / 80** | **24 / 80** | **18 / 80** |

**S2 Table.** Patient-reported assessment of generic scars (malar scar) using the POSAS 3.0 scale at baseline, post-treatment, and follow-up. Individual items are scored from 1 (no impairment/normal) to 5 (severely affected), with the total score representing the sum of 16 items (maximum 80).

**S3 Table. POSAS 3.0 Observer Linear scar: Itemized Assessment for the frontal scar.**

**S3 Table.** Observer assessment of linear scars (frontal scar) using the POSAS 3.0 scale at baseline, post-treatment, and 6-month follow-up. Scores for each item range from 1 (normal skin) to 5 (severely affected), with the total score calculated as the sum of nine items (maximum 45).

| **Items (Scale 1-5)** | **Baseline** | **Post-Treatment** | **Follow-up (6 months)** |
| --- | --- | --- | --- |
| Pigmentation | 4 (Severely/ Hyperpigmentation) | 3 (Moderately/ Mixed) | 2 (Minimally/ Hypopigmentation) |
| Vascularity | 4 (Severely/ Red + Purple) | 3 (Moderately/ Red + Purple) | 2 (Minimally/ Pale white+ Pink) |
| Surface level | 2 (Minimally/ Depressed) | 2 (Minimally/ Depressed) | 2 (Minimally/ Depressed) |
| Surface texture | 3 (Moderately/ Irregular) | 3 (Moderately/ Irregular) | 2 (Minimally/ Irregular) |
| Firmness | 3 (Moderately) | 2 (Minimally) | 2 (Minimally) |
| Adherence | 2 (Minimally) | 1 (Not) | 1 (Not) |
| Tension | 2 (Minimally) | 1 (Not) | 1 (Not) |
| Widened | 2 (Minimally) | 2 (Minimally) | 2 (Minimally) |
| Marks from surgical wound closure | 2 (Minimally) | 2 (Minimally) | 2 (Minimally) |
|  |  |  |  |
| **TOTAL (Sum 1-9)** | **24 / 45** | **19 / 45** | **16 / 45** |

**S4 Table. Posas 3.0 Patient Linear Scar: Itemized Assessment for the frontal scar.**

| **Items (Scale 1-5)** | **Baseline** | **Post-Treatment** | **Follow-up** |
| --- | --- | --- | --- |
| Colour (difference) | 2 (Little) | 2 (Little) | 1 (Not) |
| Shininess | 4 (Very) | 2 (Little) | 1 (Not) |
| Raised or Sunken (difference) | 4 (Very) | 1 (Not) | 1 (Not) |
| Hardness | 3 (Moderately) | 1 (Not) | 1 (Not) |
| Surface Irregularity | 4 (Very) | 2 (Little) | 1 (Not) |
| Edges (Stretched apart or widened) | 2 (Little) | 1 (Not) | 1 (Not) |
| Sensitive to touch | 1 (Not) | 1 (Not) | 1 (Not) |
| Less sensitive (numbness) | 1 (Not) | 1 (Not) | 1 (Not) |
| Painful | 1 (Not) | 1 (Not) | 1 (Not) |
| Shooting sensation | 1 (Not)) | 1 (Not)) | 1 (Not)) |
| Burning sensation | 1 (Not) | 1 (Not) | 1 (Not) |
| Itching | 2 (Little) | 1 (Not) | 1 (Not) |
| Tingling / Pins and needles | 1 (Not) | 1 (Not) | 1 (Not) |
| Tightness at rest | 3 (Moderately) | 1 (Not) | 1 (Not) |
| Tightness with movement | 3 (Moderately) | 1 (Not) | 1 (Not) |
| Fragility (easy breakdown) | 3 (Moderately) | 1 (Not) | 1 (Not) |
| Dryness | 2 (Little) | 1 (Not) | 1 (Not) |
| **TOTAL (Sum 1-17)** | **38 / 85** | **20 / 85** | **17 / 85** |

**S4 Table.** Patient-reported assessment of linear scars (frontal scar) using the POSAS 3.0 scale at baseline, post-treatment, and follow-up. Each item is scored from 1 (no impairment/normal) to 5 (severely affected), with the total score representing the sum of 17 items (maximum 85).
